# MicroRNA-mediated regulation of IL-10, IL-12 and TNFα gene expression in severely injured trauma patients

**DOI:** 10.1186/1757-7241-23-S2-O5

**Published:** 2015-09-11

**Authors:** Timothy F Jones, Helen C Owen, Hew DT Torrance, Negar Pirmadjid, Karim Brohi, Charles J Hinds, Michael J O'Dwyer

**Affiliations:** 1 Centre for Translational Medicine and Therapeutics, William Harvey Research Institute, Barts and the London School of Medicine and Dentistry, London, UK; 2 Centre for Trauma Sciences, Blizard Institute, Barts and the London School of Medicine and Dentistry, London, UK; 3 Adult Critical Care Unit, Royal London Hospital, Barts Health NHS Trust, London, UK

## Background

Severe trauma induces a blunted immune response associated with an enhanced susceptibility to nosocomial infections [1]. Within 2 hours of injury, expression of the prototypical anti-inflammatory cytokine, IL-10, increases whilst expression of the pro-inflammatory cytokines, TNFα and IL-12, decreases [1]. We hypothesise that microRNAs (miRs) may exacerbate this immunosuppressive gene expression pattern.

## Methods

Following ethical approval and consent, 30 ICU patients admitted following trauma and 16 healthy age and sex-matched controls were recruited. Blood samples were obtained within 2 hours of injury and at 24 hours. miRs were isolated using PAXGene (Qiagen). miRs were selected on the basis of their miRBase target prediction scores for the promoters of IL-10, IL-12 and TNFα. Six miRs selected for analysis were miR374b, -202 and 125a3p (IL-10), -410 and -21 (IL-12) and -454 (TNFα). qPCR was used to quantify candidate miRs and the results were normalised relative to small nucleolar RNAs U6/RNU44. Infections were assessed using predefined criteria [2].

## Results

Twenty three patients (77%) developed an infection, 15 (50%) were shocked (base deficit ≥ 6 mEq/L) on admission and 6 (20%) died. Within 2 hours, expressions of miR-202, -125a3p, -21 and -454 were reduced (all p<0.03) in patients compared to healthy controls. This reduction was maintained (all p<0.01) 24 hours after injury. At 24 hours, miR-202 was down-regulated (2.4-fold, p=0.01) in shocked compared to non-shocked patients. Decreased miR-374b expression on admission was associated with subsequent development of pneumonia (p=0.009).

## Conclusion

Expression of miRs complementary to cytokine promoters varies significantly following severe traumatic injury and is associated with clinical outcomes. Reduction in inhibitory miRs could partly explain increased IL-10 expression and provide a mechanistic link between severe trauma, the observed immunosuppressive phenotype and an increased incidence of nosocomial infections. In vitro studies are now needed to invoke causation.
